# Linking Atmospheric and Soil Contamination: A Comparative Study of PAHs and Metals in PM_10_ and Surface Soil near Urban Monitoring Stations

**DOI:** 10.3390/toxics13100866

**Published:** 2025-10-12

**Authors:** Nikolina Račić, Stanko Ružičić, Gordana Pehnec, Ivana Jakovljević, Zdravka Sever Štrukil, Jasmina Rinkovec, Silva Žužul, Iva Smoljo, Željka Zgorelec, Mario Lovrić

**Affiliations:** 1 Institute for Medical Research and Occupational Health, 10000 Zagreb, Croatia; nracic@imi.hr (N.R.); gpehnec@imi.hr (G.P.); ijakovljevic@imi.hr (I.J.); zsever@imi.hr (Z.S.Š.); jrinkovec@imi.hr (J.R.); szuzul@imi.hr (S.Ž.); ismoljo@imi.hr (I.S.); 2 The Lisbon Council, 1040 Brussels, Belgium; 3 Faculty of Mining, Geology and Petroleum Engineering, University of Zagreb, 10000 Zagreb, Croatia; stanko.ruzicic@rgn.unizg.hr; 4 Faculty of Agriculture, University of Zagreb, 10000 Zagreb, Croatia; zzgorelec@agr.hr; 5 Institute for Anthropological Research, 10000 Zagreb, Croatia; 6 Faculty of Food Technology Osijek, Josip Juraj Strossmayer University of Osijek, 31000 Osijek, Croatia

**Keywords:** air pollution, metals, PAHs, soil, particulate matter

## Abstract

Understanding how atmospheric pollutants interact with soil pollution is essential for assessing long-term environmental and human health risks. This study compares concentrations of polycyclic aromatic hydrocarbons (PAHs) and potentially toxic elements (PTEs) in PM_10_ and surface soil near air quality monitoring stations in Zagreb, Croatia. While previous work identified primary emission sources affecting PM_10_ composition in the area, this study extends the analysis to investigate potential pollutant transfer and accumulation in soils. Multivariate statistical tools, including correlation analysis and principal component analysis (PCA), were employed to gain a deeper understanding of the sources and behavior of pollutants. Results reveal significant correlations between air and soil concentrations for several PTEs and PAHs, particularly when air pollutant data are averaged over extended periods (up to 6 months), indicating cumulative deposition effects. Σ11PAH concentrations in soils ranged from 1.2 to 524 µg/g, while mean BaP in PM_10_ was 2.2 ng/m^3^ at traffic-affected stations. Strong positive air–soil correlations were found for Pb and Cu, whereas PAH associations strengthened at longer averaging windows (3–6 months), especially at 10 cm depth. Seasonal variations were observed, with stronger associations in autumn, reflecting intensified emissions and atmospheric conditions that facilitate pollutant transfer. PCA identified similar pollutant groupings in both air and soil matrices, suggesting familiar sources such as traffic emissions, industrial activities, and residential heating. The integrated PCA approach, which jointly analyzed air and soil pollutants, showed coherent behaviour for heavier PAHs and several PTEs (e.g., Pb, Cu), as well as divergence in more volatile or mobile species (e.g., Flu, Zn). Spatial differences among monitoring sites show localized influences on pollutant accumulation. Furthermore, this work demonstrates the value of coordinated air–soil monitoring in urban environments and provides an understanding of pollutant distributions across different components of the environment.

## 1. Introduction

Environmental pollution with polycyclic aromatic hydrocarbons (PAHs) and potentially toxic elements (PTEs) has been widely studied, particularly within individual environmental components such as air, water, or soil [1,2,3,4,5,6,7]. Numerous studies have focused on characterizing the presence, sources, and behavior of these pollutants in either the atmospheric or terrestrial environment. However, comparative analyses and correlations between compartments, particularly the complex relationships through which airborne pollutants are deposited into soil, remain relatively underexplored [1,8,9].

PAHs and PTEs are among the most prevalent pollutants found in urban air, primarily associated with particulate matter (PM), especially the PM_10_ fraction (particles with aerodynamic diameters ≤10 µm) [10,11,12]. PAHs are organic compounds composed of multiple fused aromatic rings, commonly produced during incomplete combustion of organic matter [10,13]. Major anthropogenic sources of atmospheric PAHs include motor vehicle exhaust, residential wood or coal burning, industrial processes, waste incineration, and, to a lesser extent, natural sources such as wildfires [14,15,16,17].

PTEs, including lead (Pb), cadmium (Cd), zinc (Zn), copper (Cu), arsenic (As), and nickel (Ni), can enter the atmosphere through similar pathways. In urban environments, these metals are typically emitted from traffic-related sources (e.g., fuel combustion, brake and tire wear), industrial activities, and the resuspension of contaminated dust from roads or construction sites [16,18,19,20,21].

The health impacts of PAHs and PTEs in ambient air are well established, with exposure linked to severe health conditions, including cancer, cardiovascular disease, and developmental disorders [5,22]. Many PAHs, such as benzo[a]pyrene (BaP), are classified as probable or known human carcinogens and have been associated with genotoxicity, oxidative stress, and respiratory diseases [23,24]. Chronic exposure to PM-bound metals is connected to a range of health effects, including neurotoxicity, nephrotoxicity, cardiovascular dysfunction, and cancers [25,26,27]. Due to their ability to penetrate deep into the lungs when adsorbed onto fine particles, these pollutants are considered a major public health concern, particularly in densely populated urban areas. Airborne particles serve as a transport medium for both PAHs and metals, facilitating their atmospheric dispersion and eventual deposition onto terrestrial surfaces through both wet and dry deposition processes [28,29,30]. While atmospheric monitoring provides essential information on current exposure levels and regulatory compliance, it captures only part of the environmental picture, the pollutants in active circulation. Once deposited from the atmosphere, PAHs and metals can accumulate in soils, where different dynamics govern their environmental behavior compared to when they are in the air. Soils can act as both sinks and sources of pollutants, depending on the characteristics of the pollutant, land use, and environmental conditions. PAHs in soil are often adsorbed to organic matter and exhibit slow degradation rates, particularly under anaerobic or cold conditions [6,31,32]. As a result, they can persist in the environment for extended periods and may become a source of contamination for groundwater, plants, and biota. PTEs, in contrast, are non-degradable and tend to persist indefinitely in soils [33,34]. Their mobility and bioavailability depend on several factors, including soil pH, texture, organic matter content, and redox conditions [35,36]. In contaminated soils, metals may pose risks to human health through direct contact, inhalation of resuspended dust, or uptake by crops and subsequent ingestion [35]. This highlights the role of soil as a secondary source of airborne pollutants, capable of reintroducing previously deposited metals and associated particulates back into the atmosphere [37,38]. In urban areas with high pedestrian activity, traffic, and ongoing construction, such re-emission pathways are especially relevant. Additionally, uptake by crops or homegrown vegetables in gardens near roads or industrial areas presents another route of chronic exposure through ingestion [39,40]. These mechanisms explain the importance of monitoring not just atmospheric emissions but also soil pollution and its potential to prolong or amplify human exposure, even after primary emission sources are reduced.

Although many studies have addressed the sources, concentrations, and health effects of PAHs and metals in air or soil separately, comparative investigations that assess their concentrations in both environments simultaneously are still limited. Very few studies attempt to evaluate whether the pollutant profiles in the air are reflected in the underlying soils, or whether the same emission sources contribute similarly to both media. Additionally, the degree to which current air pollution contributes to ongoing soil contamination through atmospheric deposition is not well understood. Soil sampling in the vicinity of air quality monitoring stations provides a unique opportunity to assess these relationships. If the same pollutants measured in PM_10_ are found in elevated concentrations in nearby soils, and if patterns align spatially or statistically, this can indicate either direct deposition pathways or shared source signatures. Such analyses provide valuable insights into pollutant transport and accumulation processes, as well as long-term environmental exposure risks. Croatian soils are characterized by a diverse geochemical background due to the country’s complex geological structure. According to the national Geochemical Atlas [21], concentrations of trace metals, such as Fe, Mn, Zn, Pb, and Cu, vary significantly across different regions, reflecting both natural lithological variation and anthropogenic influences. For example, elevated Zn and Pb levels in urban soils have been linked to historic industrial activities and traffic emissions, while As and Fe concentrations are more strongly controlled by geological substrates and soil-forming processes [21].

Building on previous work that identified primary sources of PAHs and metals in PM_10_ in the urban environment of Zagreb [16], this study expands the focus to include the soil component. The main objectives are

- To determine the concentrations and distribution of selected PAHs and PTEs in PM10 and surface soil samples collected near urban air quality monitoring stations over three years.
- To investigate spatial and vertical variability in soil pollutant concentrations (0–5 cm vs. 5–10 cm) and explore seasonal differences in pollutant accumulation.
- To compare pollutant profiles across air and soil compartments and assess the extent of their correlation, to evaluate atmospheric deposition as a potential pathway for soil contamination.
- To apply descriptive and multivariate statistical techniques to identify common pollution sources and better understand the inter-compartmental behavior of pollutants.

The idea of this study is to advance the understanding of pollutant behavior by integrating atmospheric and soil data, thereby contributing to a more comprehensive assessment of environmental contamination processes in urban environments. To the best of our knowledge, this is the first study conducted in this region to examine and compare PAHs and metal concentrations in both air (PM_10_) and soil, exploring their potential interactions and deposition trends.

## 2. Materials and Methods

### 2.1. Study Area and Pollutant Characterization

**Figure 1:**
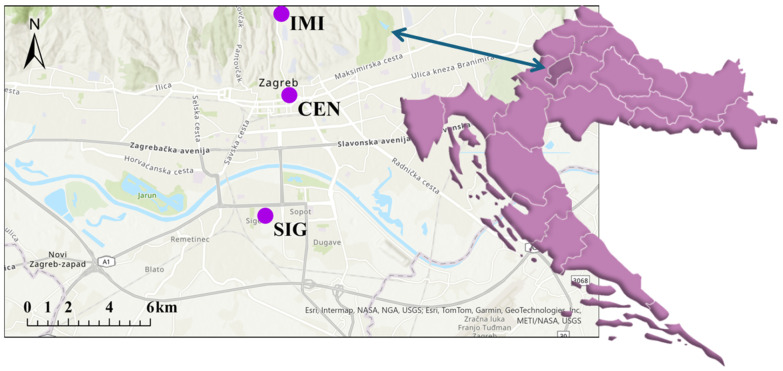
Site locations. Figure was made using ArcGIS Pro (version 3.2.0) and Adobe Illustrator (version 29.5.1). IMI (altitude 116 m a.s.l., 45°50′7′′ N, longitude 15°58′42′′ E); SIG (altitude 116 m a.s.l., latitude 45°46′25′′ N, longitude 15°59′04′′ E), CEN (altitude 113 m a.s.l., 45°48′37′′ N, longitude 15°59′4′′ E).

The city of Zagreb, situated in the northwestern part of Croatia, represents the country’s most significant urban, economic, and administrative centre. With nearly 800,000 residents, the city represents a typical urban environment, including a mix of residential, industrial, and transportation-related emission sources [41]. Seasonal variations in heating demand, connected with urban traffic, contribute to fluctuating levels of air pollutants, notably PM, PAHs, and PTEs. To capture spatial variability in urban air pollution and its potential influence on surrounding soils, three sites were selected within the city of Zagreb: Ksaverska cesta (IMI), Center (CEN), and Siget (SIG) (Figure 1).

Site selection was based on the type of monitoring station (urban background or traffic-influenced), their geographic distribution across the city, and the availability of long-term pollutant concentration data. At all three locations, there are air quality monitoring stations within Zagreb’s official air quality monitoring network, which is operated with support from the City Office for Economy, Energy, and Environmental Protection. At the IMI site, there is Ksaverska cesta monitoring station, which is categorized as an urban background site and is located in the northern residential zone of Zagreb. The station is located approximately 20 m from a road with a moderate traffic volume and is surrounded predominantly by low-rise family housing units. Heating in this area is primarily based on gas, with supplemental use of wood and oil, particularly during the winter season. The SIG site, by contrast, is located in a densely populated southern neighbourhood. The air quality monitoring station SIG is situated approximately 4 m above ground and roughly 30 m from a main road with intense traffic. The area is characterized by high-rise buildings, mostly connected to the central heating system (approximately 5 km east-southeast of SIG). Still, it is also influenced by dominant northerly winds carrying emissions from the city center and residential heating systems using various fuel types. Soil samples were collected in the immediate vicinity of the SIG station. The CEN station (air quality monitoring station Đorđićeva ulica) is positioned near the urban center, with proximity to heavily trafficked roads.

For the CEN site, PAH concentrations were not directly available at the station; therefore, data from the nearest national network air quality monitoring station (Zagreb-1) in proximity were used to represent PAH levels. The built environment in this sector consists of multi-story buildings, most of which are heated by natural gas. This location represents one of the historically most densely populated and traffic-affected parts of the city. The wider Zagreb area is situated within the southwestern part of the Pannonian Basin, characterized by Quaternary alluvial deposits overlying Neogene marls, clays, sands, and gravel layers, with local occurrences of Miocene limestones and dolomites [42]. These unconsolidated alluvial and fluvial sediments dominate the upper soil horizons across the city, providing a silicate-rich geogenic background for elements such as Fe, Mn, and Cr. Variations in soil texture and mineralogy across the urban area reflect both natural substrate heterogeneity and anthropogenic modifications due to construction, filling, and land use. This geological context is crucial for understanding the distinction between lithogenic and anthropogenic sources of trace elements in the collected soil samples [43].

At the selected monitoring stations, PM_10_ was measured daily in accordance with EU standardized reference methodologies (CEN: EN 12341:2023, EN 14902:2005 and AC:2006, EN 15549:2008, and CEN/TS 16645:2014) [44,45,46,47,48]. For this study, the period from 1 January 2018 to 31 December 2020 was chosen. During this time, continuous 24-h PM_10_ sampling was conducted using low-volume samplers (Sven Leckel, ~55 m^3^/day), with particles collected on quartz or membrane filters depending on the analytical requirements. The chemical composition of PM_10_ was assessed through analysis of selected trace metals and PAHs, including those not included in routine monitoring. Targeted metals included arsenic (As), cadmium (Cd), lead (Pb), manganese (Mn), iron (Fe), copper (Cu), and zinc (Zn). At the same time, the PAH fraction comprised benzo[a]pyrene (BaP), benzo[a]anthracene (BaA), benzo[ghi]perylene (BghiP), dibenzo[ah]anthracene (DahA), chrysene (Chry), benzo[k]fluoranthene (BkF), benzo[b]fluoranthene (BbF), benzo[j]fluoranthene (BjF), fluoranthene (Flu), pyrene (Pyr), and indeno[1,2,3-cd]pyren (IP). PAH compounds were extracted from quartz filters using ultrasonic-assisted extraction with a cyclohexane-toluene solvent mixture. Following centrifugation and evaporation, the extracts were reconstituted in acetonitrile and analyzed via high-performance liquid chromatography (HPLC) using an Agilent 1260 Infinity system (Agilent Technology, Santa Clara, CA, USA) equipped with a fluorescence detector, according to protocols adapted from Jakovljević et al. [49]. Metal concentrations were determined after microwave-assisted acid digestion (Ultraclave IV, Milestone Srl) of the filters, followed by analysis using inductively coupled plasma mass spectrometry (ICP-MS; Agilent 7500cx, Agilent Technology, Santa Clara, CA, USA), as described in Beslic et al. [18]. The monitoring stations and analysis were previously described in [50,51,52,53,54].

In parallel with air monitoring activities, surface soil samples were collected near the three air quality monitoring stations over three years (2018–2020). Sampling was carried out twice annually, in spring and autumn, to capture potential seasonal variations in pollutant deposition and accumulation. At each site, soil was sampled at two depths: 0–5 cm and 5–10 cm, targeting layers most influenced by atmospheric deposition. Composite samples were formed by combining multiple subsamples within a defined radius of each station to ensure spatial representativeness. Five individual subsamples were collected at each depth and then homogenized to form one composite sample per depth and season. This procedure was repeated at all three stations during spring and autumn campaigns, yielding a total of 36 composite samples (3 stations × 2 depths × 2 seasons × 3 years). The collected soil was air-dried, homogenized, and before chemical analysis, the samples were finely ground using a Retsch RS 200 vibratory disc mill (Retsch GmbH, Haan, Germany) to achieve uniform particle size and analytical consistency. Metal concentrations were then determined using a portable X-ray fluorescence (XRF) analyzer (HITACHI X-MET8000 Expert GEO, Hitachi High-Tech Analytical Science, Oxford, UK), which enables rapid, non-destructive elemental analysis of prepared soil samples. An Accelerated Solvent Extraction system (ASE 350, Dionex, Thermo Fisher Scientific, Sunnyvale, CA, USA) was used to extract PAHs from soil samples. Extractions were performed at 1500 psi and 125 °C, using a flush volume of 70% of the extraction cell volume. A 1:1 (*v*/*v*) mixture of n-hexane and acetone was used as the extraction solvent. The final extracts were concentrated to 1 mL, and 10 ng of the internal standard, perylene-d_12_, was added to each sample. Quantification of 11 PAHs was carried out using gas chromatography coupled with tandem mass spectrometry (GC–MS/MS; Agilent 7890B–7000C, Agilent Technology, Santa Clara, CA, USA) in multiple reaction monitoring (MRM) mode. The GC was equipped with a DB-EUPAH capillary column (20 m × 180 µm i.d., 0.14 µm film thickness), using helium as the carrier gas.

All analytical procedures were performed under strict QA/QC protocols. For PM_10_ samples, calibration was carried out using commercial standards, and certified reference materials (e.g., NIST SRM 1649a for PAHs, ERM-CZ120 for metals) were analyzed in parallel. Calibration curve linearity, recovery, and repeatability were checked routinely, with recovery rates for both PAHs and metals falling within 85–115%. The same QA/QC principles were applied to soil PAH analysis. Calibration standards and surrogate spikes were used to assess method performance, with recoveries within accepted ranges. All concentrations were corrected for recovery. Procedural, laboratory, and field blanks were included to monitor potential contamination, and replicate analyses confirmed reproducibility.

### 2.2. Data Processing and Statistical Analysis

The dataset for this study comprises daily records of pollution data from three air quality monitoring stations as well as data from soil samples. The analysis was conducted using the Python programming language (www.python.org, v3.10). Air pollutant concentrations were aggregated by calculating median values over defined temporal windows of 1, 2, and 3 months before each soil sampling season to capture potential lagged deposition effects. Soil pollutant concentrations were transformed to a long format and harmonized with air data by matching pollutant type, station, season, and year. Spearman rank correlation coefficients were then computed for each pollutant to assess the relationships between airborne and soil-bound concentrations, focusing on the matched sampling periods and locations. This approach enabled the examination of temporal and spatial relationships between air and soil contamination, providing information about pollutant behavior across different environmental media.

To explore pollutant sources and trends, principal component analysis (PCA) was conducted separately on the standardized datasets of air and soil pollutants. PCA helped identify groups of pollutants with common variance patterns, which can be interpreted as indicative of shared sources or similar environmental behavior in different environmental matrices. To explore the relationships and grouping between individual air and soil pollutants, we performed a PCA using concentration data from paired air and topsoil (5 cm depth) samples. Before PCA, non-numeric variables and metadata columns (e.g., station, year, medium) were excluded. To compare pollutant behavior across media, the dataset was transposed so that each pollutant became a separate row (observation) and each sample became a variable. The transposed data matrix was then standardized using z-score normalization, ensuring each pollutant was centered and scaled to unit variance. PCA was performed on the standardized matrix using the scikit-learn PCA implementation. PCA results were visualized in a two-dimensional score plot, with each point representing a pollutant-medium pair. Because soils integrate inputs over longer periods than instantaneous air concentrations, we paired each soil campaign with rolling 1-, 3-, and 6-month medians of prior airborne concentrations to capture seasonal-scale deposition signals. The 0–5 cm and 5–10 cm depths were analyzed to differentiate recent vs. historical accumulation signatures.

## 3. Results and Discussion

### 3.1. General Pollution Levels in Air and Soil

Across the three monitoring stations in Zagreb, PAH and metal concentrations in PM_10_ showed spatial variability. For the overall period, SIG exhibited the highest mean concentrations for all PAHs and metals, including BaP (2.15 ng/m^3^), BghiP (2.14 ng/m^3^), and BbF (2.44 ng/m^3^). IMI and CEN stations generally showed lower PAH levels, although CEN had notable levels of BaP (1.25 ng/m^3^) and BghiP (1.26 ng/m^3^), suggesting the impact of urban central emissions. Similar trends were observed for PTEs: SIG reported the highest concentrations of Fe (543.51 ng/m^3^), Zn (28.14 ng/m^3^), and Cu (17.37 ng/m^3^), which are likely linked to traffic-related sources, such as brake and tire wear. In contrast, the IMI site showed the lowest levels for most metals, possibly due to lower direct emissions or dispersion effects.

Based on the dataset of 11 PAH compounds measured in topsoil (0–5 cm) and subsoil (5–10 cm) samples collected in spring and autumn of 2018–2020 at three stations, the results show notable spatial, seasonal, and vertical trends (Figure S1 in Supplementary Materials). To our knowledge, these are the first systematically collected and analysed data on PAHs in soils from Zagreb and among the few such datasets available for Croatia. The Andrija Štampar Teaching Institute of Public Health occasionally conducts measurements of PAHs and metals in Zagreb soils as part of its environmental monitoring activities, with results accessible via the City of Zagreb’s Eco Map portal (https://ekokartazagreb.stampar.hr/, accessed on 10 August 2025). However, this monitoring is occasional rather than systematic, is conducted at varying locations without repeated sampling at the same sites, and the data is not regularly processed or published in the scientific literature, serving instead as public information on whether guideline values are met. In this study, Σ11PAH concentrations in Zagreb urban soils ranged from 1.23 to 524,098 ng/g, with BaP between 0.05 and 20,375 ng/g. These values align with the global urban soil ranges summarized by Wilcke [55], where background urban sites typically contain 10^2^–10^5^ ng/g ΣPAH, and industrial hotspots can exceed 10^6^ ng/g. Similarly, Li et al. [56] reported urban soil ΣPAHs spanning <100 to >106 ng/g, with traffic and residential combustion as dominant sources. Compared to regional data, Zagreb soils overlap with the lower to mid-range of values from Novi Sad, Serbia (180–24,500 ng/g Σ16PAH [57]). Still, they are substantially below the high contamination levels found in Glasgow, Torino, and Ljubljana [6]. Overall, the Zagreb results indicate moderate urban PAH contamination, consistent with the ranges reported for cities influenced primarily by traffic and domestic heating emissions, rather than by heavy industrial activity.

**Figure 2:**
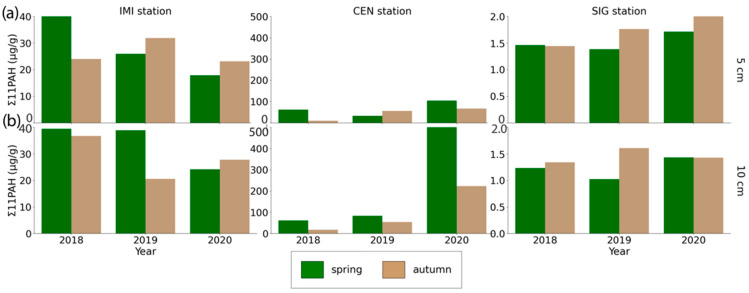
Annual variation (*x*-axis, 2018–2020) of sum PAH concentrations in soil at depths (**a**) 0–5 cm; (**b**) 5–10 cm at monitoring sites IMI, CEN, and SIG, in µg/g, mean for both seasons.

Across sites, Σ11PAH concentrations showed both seasonal and depth-related differences (Figure 2). At CEN, values were higher at 10 cm than at 5 cm for most seasons. At IMI, seasonal means were more balanced, with slightly higher values at 10 cm in both spring and autumn, but no major depth effect was observed. At SIG, concentrations remained uniformly low regardless of depth or season, with only minor variation between 5 cm and 10 cm layers.

The IMI site exhibited moderately high PAH concentrations, particularly in spring 2018 at a 5 cm depth (Sum11PAH = 46.448 µg/g), with a dominance of BaA (10.433 µg/g), Chry (8.751 µg/g), and BbF (8.394 µg/g). Over time, concentrations at IMI declined, particularly by the spring of 2020, when sum11PAH dropped to 17.884 µg/g at 5 cm. The CEN station shows the highest PAH contamination, particularly in spring 2020, with elevated values at 10 cm depth (Sum11PAH = 524.098 µg/g), driven by extremely high BaA (142.913 µg/g), Pyr (94.192 µg/g), Chry (74.555 µg/g), and BbF (72.905 µg/g). Even at 5 cm depth, spring 2020 values reached 105.714 µg/g, again highlighting BbF, BaA, and Chry as key contributors.

The exceptionally high PAH concentrations observed at CEN in spring 2020 could reflect a combination of local emission events and environmental disturbances. In particular, the March 2020 Zagreb earthquake and subsequent clean-up activities may have contributed to resuspension of contaminated dust in the city center, while increased residential heating during the unusually cold early spring period may have further elevated emissions [53]. Episodic inputs of this kind are consistent with the observed sharp interannual variability at CEN but not at other sites. Autumn levels were generally lower, but still substantial, e.g., 67.838 µg/g at 5 cm in autumn 2020. In contrast, the SIG station consistently exhibited very low PAH concentrations. For example, spring 2018 values were only 1.502 µg/g at 5 cm and 1.276 µg/g at 10 cm. Even by the 2020 autumn, concentrations remained low at 2.086 µg/g and 1.475 µg/g at 5 and 10 cm, respectively.

**Figure 3:**
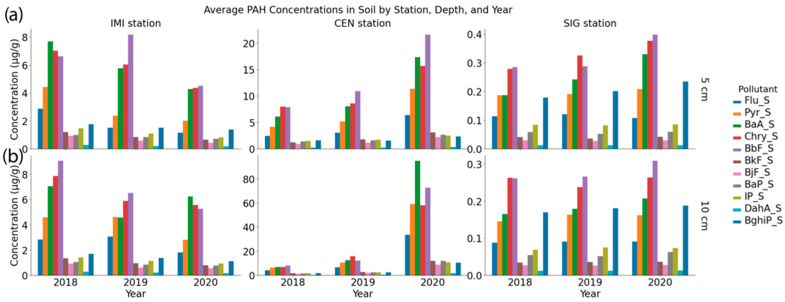
Annual variation (*x*-axis, 2018–2020) of PAH concentrations in soil at depths (**a**) 0–5 cm; (**b**) 5–10 cm at monitoring sites IMI, CEN, and SIG, in µg/g, mean for both seasons.

In Figure S1 in the Supplementary Materials, seasonal differences are not consistent: in some years and stations, spring values are higher (e.g., IMI 2018, CEN 2020), while in others, autumn is higher or the difference is minimal. Therefore, the overall pattern is better represented by averaging concentrations by season (Figure 3), which reveals a clear decreasing trend at IMI, a pronounced increase at CEN (driven mainly by 2020), and persistently low but slightly increasing levels at the SIG site. This pattern aligns with previous studies, which show that in urban soils, interannual variability and episodic inputs (such as heating/combustion, traffic, and atmospheric deposition) often override a stable seasonal cycle. Winter and spring inputs are frequently elevated but are strongly influenced by local sources and meteorological conditions. The dominance of 4–6-ring PAHs and the observed concentration ranges are comparable to other moderately impacted European cities [6], supporting the interpretation that local sources and atmospheric deposition are the primary drivers of variability. These findings revealed the impact of site-specific characteristics, seasonal atmospheric inputs, and pollutant mobility in explaining PAH distribution in soils.

**Figure 4:**
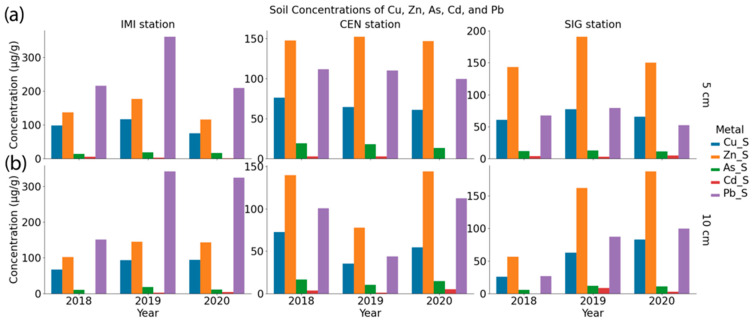
Annual variation (*x*-axis, 2018–2020) of metal concentrations in soil at depths (**a**) 0–5 cm; (**b**) 5–10 cm at monitoring sites IMI, CEN, and SIG, in µg/g, mean for both seasons.

The concentrations of selected metals (Cu, Zn, As, Cd, and Pb) in soil varied considerably across stations, depths, and sampling periods (Figure 4).

Among all metals, iron (Fe) shows the highest concentrations, with values ranging from 8950 µg/g to 54,215 µg/g, depending on the station and depth. Mn concentrations also showed significant variability, spanning from 212 µg/g to over 1130 µg/g, with generally higher values observed in CEN and IMI stations compared to SIG. Cu and Zn concentrations were highest at SIG, where Zn reached up to 219 µg/g and Cu exceeded 120 µg/g in some samples. Arsenic levels ranged from 4.3 µg/g to approximately 22 µg/g, indicating moderate fluctuations across locations and depths. Cd, while generally present at low concentrations (often below five µg/g), occasionally peaked at 8.5 µg/g, particularly at SIG in 2019. Lead concentrations showed a broad range (from 21 µg/g to over 388 µg/g), with particularly elevated values observed at IMI in 2019 and CEN in 2020. The IMI peak likely reflects dust from demolition and roadworks for the new Institute building near the station, while the CEN peak may be linked to dust resuspension after the March 2020 Zagreb earthquake and post-quake clean-up in the city centre [58]. These values are comparable to, or exceed, those previously reported for Zagreb soils, where Pb ranged 1.5–139 µg/g, Zn 15–277 µg/g, Cu 4–183 µg/g, Mn 79–1282 µg/g, and Fe 5.8–51.8 g/kg, with spatial patterns reflecting both anthropogenic influences (traffic emissions, industrial activities, waste disposal) and lithogenic controls [59]. Other studies in the Zagreb region similarly identified Zn, Pb, Cd, and Cu as markers of urban and industrial contamination, with short-range variability linked to local emission sources and long-range variability reflecting geological background [60]. Elevated levels of several metals in earlier surveys were also associated with traffic, industrial history, and riverine deposition from the Sava [54]. Overall, higher concentrations of several metals (notably Cu, Zn, and Pb) tended to occur in topsoil (5 cm) and during spring campaigns, suggesting possible influences from seasonal deposition and proximity to anthropogenic sources (Figures S2–S4 in Supplementary Materials).

### 3.2. Relationships Between Air and Soil Pollutants

**Figure 5:**
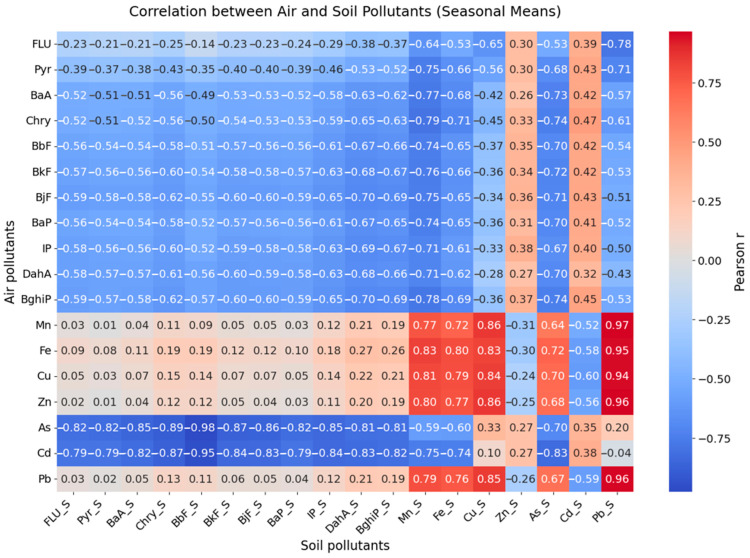
Correlation heatmap between air and soil pollutants (air: seasonal mean concentrations; soil: mean across both seasons and depths).

As a first step, the relationships between air and soil concentrations of selected PAHs and trace metals were investigated by correlating seasonal soil pollutant levels with median air pollutant concentrations for that same season. The heatmap visualization of all pollutants indicated some clustering of metals with positive cross-correlations, while many PAHs exhibited negative or negligible correlations between air and soil phases (Figure 5). PTEs such as Pb, Mn, Fe, As, and Cu showed strong positive correlations between air and soil, suggesting that they may share similar seasonal emission and deposition patterns, potentially resulting from traffic, construction activities, and the resuspension of contaminated dust. However, As and Cd exhibited negative correlations between air and soil concentrations, indicating possible differences in source profiles, atmospheric behavior, or soil retention dynamics. The negative correlations observed for several PAHs between air and soil likely reflect differences in environmental behavior compared to those of trace metals. Many PAHs are semi-volatile and subject to bidirectional exchange between soil and atmosphere, especially under higher temperatures, which can result in re-emission rather than net accumulation. Atmospheric degradation (e.g., photolysis and reactions with OH radicals or ozone) further reduces the fraction available for deposition. In soil, microbial degradation and sorption–desorption dynamics also influence persistence; lighter PAHs (e.g., Flu, Pyr) are more mobile and degrade faster, leading to weaker or even inverse associations with recent airborne levels. Moreover, site-specific soil properties, such as organic matter content, texture, and pH, affect retention, with soils of lower organic carbon content showing reduced PAH binding and greater losses. Together, these processes can explain why metals mostly tend to show positive air–soil correlations, while PAHs often display weaker or negative relationships. This suggests that complex deposition, degradation, or source factors influence pollutant behavior in soil [61]. Comparable findings have been reported in other European urban soil studies, where self-organizing map analyses highlighted the distinct clustering of lithogenic versus traffic-related elements and underscored the complex behavior of PAHs in surface soils [62].

The Spearman correlation analysis revealed an association between air and soil pollutant concentrations at two soil depths (0–5 cm and 5–10 cm, Table S1 in Supplementary Materials). At 0–5 cm depth, the strongest positive correlations were observed for Pb (R = 0.94, *p* = 0.005), Cd (R = 0.88, *p* = 0.020), and Mn (R = 0.77, *p* = 0.072), indicating a close association between their airborne concentrations and accumulation in the surface soil layer. Fe (R = 0.77) and Cu (R = 0.71) also showed moderately strong positive correlations. At the same time, most PAHs displayed moderate to strong negative correlations (e.g., Flu, DahA), suggesting different environmental behaviors or source–sink dynamics for these compounds. At 5–10 cm depth, Pb (R = 0.83, *p* = 0.042), Mn (R = 0.94, *p* = 0.005), and Fe (R = 0.89, *p* = 0.019) remained strongly positively correlated with their airborne concentrations. For PAHs, negative correlations persisted but were generally weaker than in the surface layer, indicating attenuation of the air–soil linkage with depth, likely due to the reduced influence of recent deposition and the greater dominance of historical accumulation or degradation processes. These findings are consistent with other urban studies, which show trace metal accumulation in topsoil, particularly Pb and Zn, due to legacy emissions and their affinity for organic matter and clays [63,64].

As a next step, the analysis of Spearman correlation coefficients between airborne pollutant concentrations and soil pollutant levels across different averaging windows (1, 3, and 6 months before soil sampling, Table S2 in Supplementary Materials) reveals important information about the temporal dynamics of pollutant deposition and accumulation in soil. The results indicate that for several pollutants, particularly PTEs such as Pb and Cu, correlations with soil concentrations are already high at 1 month and remain consistently strong or slightly decrease with longer averaging windows. Pb, for example, shows robust positive correlations across all windows (R = 0.825 at 5 cm and R = 0.727 at 10 cm for the 1-month average), confirming that soil acts as a cumulative sink for airborne Pb over extended periods. In contrast, most PAHs display weak or inconsistent correlations at 1 month but strengthen markedly over 3–6 months, particularly at 10 cm depth for compounds such as Chry, BaP, BbF, and Flu (R often > 0.6, *p* < 0.05), suggesting that seasonal-scale integration better captures their deposition into subsurface soil layers. The generally weaker correlations at short windows likely reflect the influence of episodic events, seasonal variability, atmospheric degradation, and differences in deposition pathways. These findings support the view that for persistent metals, both short- and long-term airborne levels are relevant to soil contamination, whereas for PAHs, longer averaging periods provide more meaningful soil–air relationships due to slower accumulation and greater atmospheric variability. Soil typically integrates pollutants over time due to limited mobility and slower degradation compared to the atmosphere [65]. Not all pollutants respond similarly to the averaging window length. Some PAHs, such as Pyr, exhibit weak or even slightly negative correlations at shorter windows but improve modestly at longer windows, suggesting that episodic events or seasonal variations may influence atmospheric concentrations, complicating short-term relationships. The weaker correlations at shorter windows may also result from differential atmospheric transport, degradation rates, or variable emission sources. For metals with relatively stable emissions and slow soil dynamics, longer averaging windows yield more meaningful correlations. Conversely, for more volatile or episodic compounds, additional factors beyond simple temporal averaging may better explain soil–air relationships.

To explore potential seasonal differences, Spearman correlations were calculated separately for the spring and autumn seasons, for both soil depths (Table S3 in the Supplementary Materials). Using the 6-month window, autumn shows strong positive soil–air correlations for metals, led by Pb (R = 0.900, *p* = 0.001 at 5 cm; R = 0.767, *p* = 0.016 at 10 cm) and Cu at 5 cm (R = 0.750, *p* = 0.020), with Fe and Mn positive but borderline at 10 cm (Fe R = 0.633, *p* = 0.067; Mn R = 0.650, *p* = 0.058); Arsenic is strongly negative at 5 cm (R = −0.783, *p* = 0.013), and many PAHs are negative (e.g., IP R = −0.700, *p* = 0.036 at 5 cm; R = −0.650, *p* = 0.058 at 10 cm; BjF R = −0.650, *p* = 0.058 at 5 cm; BkF R = −0.636, *p* = 0.066 at 5 cm). In spring, PAHs remain mostly negative at 5 cm, BghiP (R = −0.733, *p* = 0.025), BaP (R = −0.667, *p* = 0.050), BkF (R = −0.667, *p* = 0.050), BjF (R = −0.700, *p* = 0.036), while metals are mixed: Pb stays positive but weaker (R = 0.650, *p* = 0.058 at 5 cm; R = 0.467, *p* = 0.205 at 10 cm), Fe is modest (R = 0.550, *p* = 0.125 at 5 cm), and Cu is weak (R = 0.300, *p* = 0.433 at 5 cm). Using a 6-month window, the seasonal analysis lines up with pollutant chemistry. Metals, especially Pb and partly Cu, exhibit a stable positive soil–air relationship, strongest in autumn, reflecting their persistence and strong sorption that drives long-term accumulation into deeper layers. PAHs, being semi-volatile and more reactive, show mostly negative within-season correlations, strongest at 5 cm, due to faster turnover, degradation, and temperature-driven exchange. This explains why earlier non-seasonal positive PAH signals at 10 cm likely came from between-season contrasts rather than true within-season co-variation.

The PCA biplots for both soil (mean for all periods of measurement and both depths) and air (PM_10_, full dataset) data across the IMI, CEN, and SIG stations show clear groupings of PAHs and metals that suggest common sources and transport pathways (Figure S5 in Supplementary Materials). In soil, PAHs cluster together, likely reflecting combustion or industrial emissions, while metals form a distinct group indicating shared origins such as traffic emissions or geogenic background. The air PCA similarly shows separation between PAHs and metals, with consistent clustering of metals and PAHs across stations, confirming their atmospheric sources are primarily related to combustion processes. The alignment of pollutant groupings between soil and air suggests that atmospheric deposition is the primary mechanism for transferring airborne contaminants to soils. Although the patterns are generally coherent, differences in pollutant loadings and PCA scores across stations indicate that site-specific factors influence pollutant distribution, such as local emission sources or soil properties. Overall, these PCA results support the impact of atmospheric pollution as a soil contaminant and emphasize the value of integrated air and soil monitoring for understanding pollutant sources and dynamics in the region.

To further explore sources, separate PCA analyses were conducted for PAHs and for metals across all stations. These individual biplots (Figures S6 and S7 in the Supplementary Materials) allow more straightforward interpretation of pollutant groupings, highlighting how specific PAHs or metals contribute to variability across sites. The PCA biplots for soil PAHs reveal both common trends and compound-specific variations across the IMI, CEN, and SIG stations. At IMI, most PAHs, such as BbF, BkF, DahA, and IP, cluster tightly, suggesting a common source likely related to combustion. In contrast, BaA and BghiP project more distinctly, indicating slightly different behavior or source influence. At CEN, the PAHs group is even more closely aligned along PC1, especially BaP, DahA, and IP, highlighting a dominant shared source, possibly high-temperature combustion or industrial emissions. Chry and BbF show moderate separation, suggesting some variability in input. Similar source-related PAH patterns have also been reported in previous studies from the Zagreb area [16,50,66].

In contrast, SIG shows the most dispersion among PAHs: BaA, Chry, BghiP, and BbF align with PC2, while Flu, Pyr, DahA, and BkF deviate downward, implying mixed sources or altered retention in soil, potentially due to local soil or emission characteristics. These differences suggest that while most PAHs are co-emitted and behave similarly, certain compounds exhibit site-specific behaviors likely driven by localized conditions or additional sources. The PCA biplots for metals in soil show consistent grouping patterns across stations, with some metal-specific distinctions. At IMI, Zn, Cu, Fe, and Pb cluster along PC1, suggesting a shared origin, likely from traffic or urban emissions. In contrast, As and Mn show stronger projections on PC2, indicating different geochemical behaviors or additional sources. At CEN, metals are tightly grouped, especially Pb and Zn, indicating a dominant, likely anthropogenic source; As, however, separates clearly, suggesting a possible geogenic origin. At SIG, most metals form a compact group along PC1, confirming common urban sources, while As and Pb display different orientations, possibly reflecting site-specific inputs or soil retention differences. Overall, the patterns suggest that while most metals in soil originate from shared sources, As and, to some extent, Pb exhibit unique spatial behavior across stations.

The PCA results for both PAHs and metals in air show consistent groupings indicative of shared sources. High molecular weight PAHs such as BaP, BbF, BkF, DahA, and BghiP cluster tightly at all stations, suggesting dominant sources from combustion processes (e.g., traffic, heating), while lighter PAHs (Flu, Pyr) show separation along PC2, especially at CEN, indicating variable atmospheric behavior [16]. In contrast, the PCA of metals shows more pronounced inter-station differences. At IMI, Mn and Fe are clearly separated from other metals along PC2, indicating a more substantial contribution from crustal or industrial sources. In contrast, Pb, Zn, Cd, and Cu cluster together along PC1, suggesting a connection to traffic-related emissions. At CEN, metals are more tightly grouped, but As and Cu stand apart with strong PC2 loading, likely reflecting industrial input or long-range transport. SIG exhibits a similar grouping structure to CEN, but with more dispersed (wider) loading vectors, indicating that the same types of sources are present, albeit with greater variation in their relative contributions or influence. These results align with earlier studies in Zagreb, which identified vehicular emissions and residential heating as the leading contributors to airborne PAHs and metals [16,51]. They are also consistent with broader European evidence, which shows that Ni, Cr, and Co are primarily controlled by geological background. In contrast, Cd, Zn, and Cu tend to reflect anthropogenic enrichment and higher mobility [66,67].

**Figure 6:**
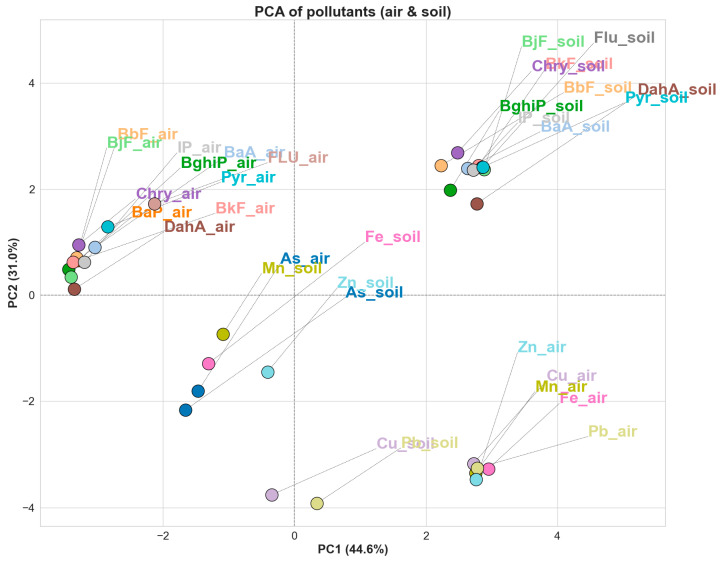
PCA score plot of air and soil pollutants, based on standardized concentrations across all samples.

To explore the relationships between pollutant concentrations in air and soil, a modified PCA was performed on a transposed dataset, where pollutants were treated as observations. In this approach, the average concentrations of PAHs and trace metals in both air and soil were used to construct a matrix, where each row corresponds to a pollutant and each column represents a sampling event. This PCA (Figure 6) illustrates how individual pollutants behave across various environmental media and sampling conditions, enabling the visual clustering of pollutants that exhibit similar environmental distribution patterns. Unlike conventional PCA, which groups similar samples, this approach enables the direct comparison of air–soil pairs of the same compound, thereby explaining their environmental stability, partitioning behavior, and potential common emission sources.

The results show that many PAHs form different air and soil pairs in the PCA space, suggesting consistent behavior across environmental components. Heavier PAHs are typically associated with combustion-related emissions such as vehicle exhaust, residential heating, and industrial processes, and are strongly particle-bound. Their semi-volatile nature allows them to travel through the atmosphere adsorbed onto fine particulate matter (PM), before depositing and accumulating in topsoil, where they persist due to low water solubility and slow degradation rates. Flu and Pyr exhibit an interesting divergence in the media. In the air, they are spatially separated from the cluster of heavier PAHs (like BaA, BbF, BkF, etc.), likely due to their higher volatility, lower molecular weight, and greater tendency to remain in the gas phase. This reduces their association with particle-bound transport and makes them less likely to behave like the heavier PAHs in atmospheric conditions. However, in soil, their positioning within or near the PAH cluster suggests that once deposited, they can accumulate similarly to heavier compounds, possibly due to adsorption to soil organic matter or repeated seasonal deposition. This asymmetry between air and soil behavior reflects efficient atmospheric transport and episodic deposition on one hand, and strong sorption and environmental persistence in soil on the other.

For trace metals, the PCA indicates varied behavior between elements. Fe and Mn, both of which are naturally abundant crustal elements, show relatively balanced air–soil associations. In urban settings, they are also connected to resuspended road dust and industrial activity. Their proximity in PCA space suggests both geogenic background and anthropogenic enhancement. Pb and Cu also demonstrate coherent air–soil clustering, consistent with their well-established links to traffic-related sources, particularly brake and tire wear. These metals, often associated with finer particles, can be efficiently deposited in soil and are relatively stable, especially in surface layers where they bind to organic matter and clays. Elevated levels of Pb and Cu in Croatian urban soils are clearly associated with anthropogenic influences, especially in high-traffic zones such as Zagreb [21].

Zn, on the other hand, shows marked divergence between its air and soil forms. In the air, Zn is often associated with fine particulate matter emitted from tire wear, lubricating oils, and galvanized surfaces, making it relatively mobile and subject to short-term variability driven by traffic intensity and meteorological conditions. In contrast, Zn in soil may reflect a combination of recent deposition and legacy contamination. Still, its mobility in the soil environment is higher than that of Pb or Cu, particularly in acidic or organic-poor soils. This can result in less retention and spatial variability in soil concentrations.

Additionally, Zn’s tendency to leach or redistribute within the soil profile may further decouple its air–soil association compared to more strongly bound metals, such as Pb and Cu. Arsenic shows strong proximity between its air and soil forms in the PCA space, suggesting similar sources and stable environmental partitioning. This coherence may reflect widespread regional emissions from combustion, with efficient deposition and retention in soil. Arsenic’s behavior in soil is also influenced by pH and redox conditions, which can stabilize it and reduce its mobility, allowing it to persist and mirror airborne inputs.

Overall, this PCA confirms that while PAHs, especially heavier ones, tend to display coherent behavior across air and soil due to their shared emission sources and stable sorption to particles, trace metals exhibit more variable relations depending on their source, mobility, atmospheric reactivity, and soil retention characteristics. Soil often reflects long-term cumulative deposition and retention, whereas air may represent more recent or transient emissions. This transposed PCA approach provides a novel perspective for simultaneously visualizing pollutant behavior across environmental compartments, underscoring the importance of integrated monitoring in evaluating pollutant fate and transport. Future studies should include long-term and seasonal soil monitoring, as well as direct measurements of particulate and total deposition, to better understand pollutant transfer. Expanding to other media, such as vegetation or dust, and integrating modeling could help assess accumulation trends and long-term risks.

PCA biplots show a clear separation between soil and PM_10_ at all stations, indicating limited cross-compartment coherence for PAHs; even heavier PAHs cluster primarily within each compartment rather than jointly across air and soil. Metals display mixed patterns consistent with source heterogeneity, mobility, and soil retention, but again do not form a unified air–soil cluster. Interpretations have several limitations: the air side relies only on PM_10_ (appropriate for health relevance and data availability, but less direct for soil transfer), no deposition fluxes were measured, and site histories of soil disturbance (e.g., excavation or imported material) are unknown. Another limitation is the short atmospheric record compared with the longer integration times of soils. This restricts assessment of decadal accumulation, though the use of multi-month air averages and two soil depths partly addresses recent deposition signals. Longer air records and deposition flux data would enhance the evaluation of long-term trends, which can be applied and expanded upon in future research.

Despite these limitations, the dataset provides the first published PAH measurements in Zagreb with systematic, multi-year seasonal soil sampling, offering a valuable baseline. Future work should pair soil campaigns with deposition measurements, include other size fractions and co-located metadata on soil management, and extend to additional media (e.g., vegetation, settled dust) and process-based modeling to resolve transfer pathways and long-term accumulation.

## 4. Conclusions

In this study, concentrations of various PAHs and metals were measured in both ambient air (PM_10_) and soil samples collected across multiple monitoring stations in Zagreb, Croatia. Soil samples were collected from two depths and analyzed for the same suite of pollutants to investigate the extent and patterns of pollutant deposition and accumulation. Statistical analyses, including Spearman correlation and PCA, were applied to explore relationships between air and soil pollutant concentrations and to identify main sources.

Seasonal differences were evident, with a 6-month window, as metals, especially Pb, showed a robust air–soil correlation. In contrast, PAHs generally exhibited negative within-season correlations, which were stronger at 5 cm, indicating surface-biased, season-dependent correlations. PCA separated soil from PM_10_, with PAHs and metals clustering within each compartment rather than jointly, revealing soil’s longer-term retention versus more transient atmospheric variability. Spatial variability among monitoring stations explained localized factors affecting pollutant distributions. This study presents the first systematically collected dataset of PAH concentrations in soils of Zagreb, combined with co-located multi-year PM_10_ measurements, allowing for a direct investigation of air–soil pollutant linkages in an urban setting. Concentrations of Σ11PAHs in soils (1.2–524 µg/g) indicate moderate but site-specific contamination. In contrast, metal enrichment (Pb up to 388 µg/g, Zn up to 219 µg/g) reflects both traffic and heating sources. In PM_10_, mean BaP reached 2.15 ng/m^3^ at traffic-affected sites, with Fe, Zn, and Cu dominating the trace metal fraction. Strong seasonal and depth-dependent air–soil correlations were observed for Pb, Mn, and Cu, supporting deposition as a key transfer pathway. In contrast, PAHs showed weaker or negative short-term correlations due to their semi-volatile nature and degradation. The novelty of this work lies in linking multi-year atmospheric and soil datasets to reveal both cumulative and short-term changes in deposition signals. Future studies should extend monitoring to longer timeframes, include direct deposition fluxes and additional media (e.g., vegetation, settled dust), and apply process-based modeling to quantify pollutant transfer pathways and long-term accumulation better. Overall, this integrated approach helps in understanding pollutant transfer from air to soil and provides valuable insights for environmental monitoring, source identification, and risk assessment studies.

## Data Availability

The data that support the findings of this study are available from the corresponding author, [M.L.], upon reasonable request.

## Supplementary Materials

The following supporting information can be downloaded at https://www.mdpi.com/article/10.3390/toxics13100866/s1. Figure S1. PAH concentrations in soil by season, depth and station. Figure S2. Metals concentration in soil by season, depth and station. Figure S3. Fe concentration in soil by season, depth and station. Figure S4. Mn concentration in soil by season, depth and station. Table S1. Correlation analysis for air (PM_10_) and soil (for different depth) pollutants. Table S2. Correlation analysis for air (different time windows) and soil pollutants. Table S3. Correlation analysis for air and soil pollutants for different seasons and depths (for time windows of 6 months for air (PM_10_) pollutant concentrations). Figure S5. PCA for the soil pollutants (a) and air (PM_10_) pollutants (b) for different monitoring stations. Figure S6. PCA for PAHs and metals in soil samples for different monitoring stations (mean for both depth and periods). Figure S7. PCA for PAHs and metals air samples for different monitoring stations.
