# Supplementary material for: Linking Atmospheric and Soil Contamination: A Comparative Study of PAHs and Metals in PM10 and Surface Soil near Urban Monitoring Stations
Source: Toxics. 2025 Oct 12;13(10):866. doi: 10.3390/toxics13100866 (PMC12567915; doi:10.3390/toxics13100866)
Supplement: Supplementary file 1 [file toxics-13-00866-s001.zip › toxics-3898102-supplementary.pdf]

SUPPLEMENTARY MATERIAL

Linking Atmospheric and Soil Contamination: A Comparative Study of PAHs and Metals in PM_{10} and Surface Soil near Urban Monitoring Stations

Nikolina Račić [1,2], Stanko Ružičić [3], Gordana Pehnec [1], Ivana Jakovljević [1], Zdravka Sever Štrukil [1], Jasmina Rinkovec [1], Silva Žužul [1], Iva Smoljo [1], Željka Zgorelec [4] and Mario Lovrić [2,5,6,*]

1 Institute for Medical Research and Occupational Health, 10000 Zagreb, Croatia; nracic@imi.hr (N.R.); gpehnec@imi.hr (G.P.); ijakovljevic@imi.hr (I.J.); zsever@imi.hr (Z.S.Š.); jrinkovec@imi.hr (J.R.); szuzul@imi.hr (S.Ž.); ismoljo@imi.hr (I.S.)
2 The Lisbon Council, 1040 Brussels, Belgium
3 Faculty of Mining, Geology and Petroleum Engineering, 10000 Zagreb, Croatia; stanko.ruzicic@rgn.unizg.hr
4 Faculty of Agriculture, University of Zagreb, 10000 Zagreb, Croatia; zzgorelec@agr.hr
5 Institute for Anthropological Research, 10000 Zagreb, Croatia
6 Faculty of Food Technology Osijek, Josip Juraj Strossmayer University of Osijek, 31000 Osijek, Croatia
* Correspondence: mario.lovric@inantro.hr

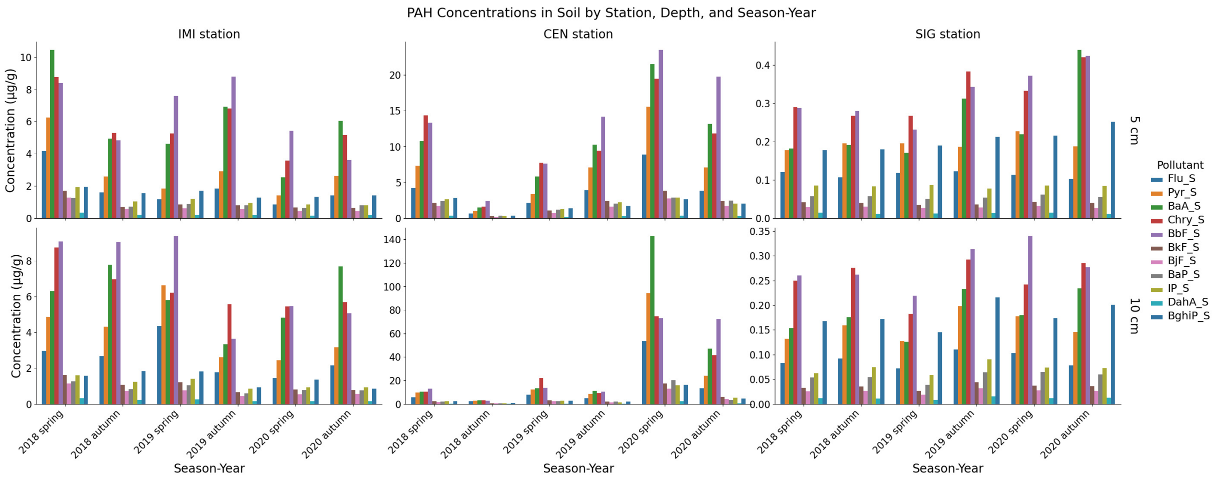

Figure S1. PAH concentrations in soil by season, depth and station

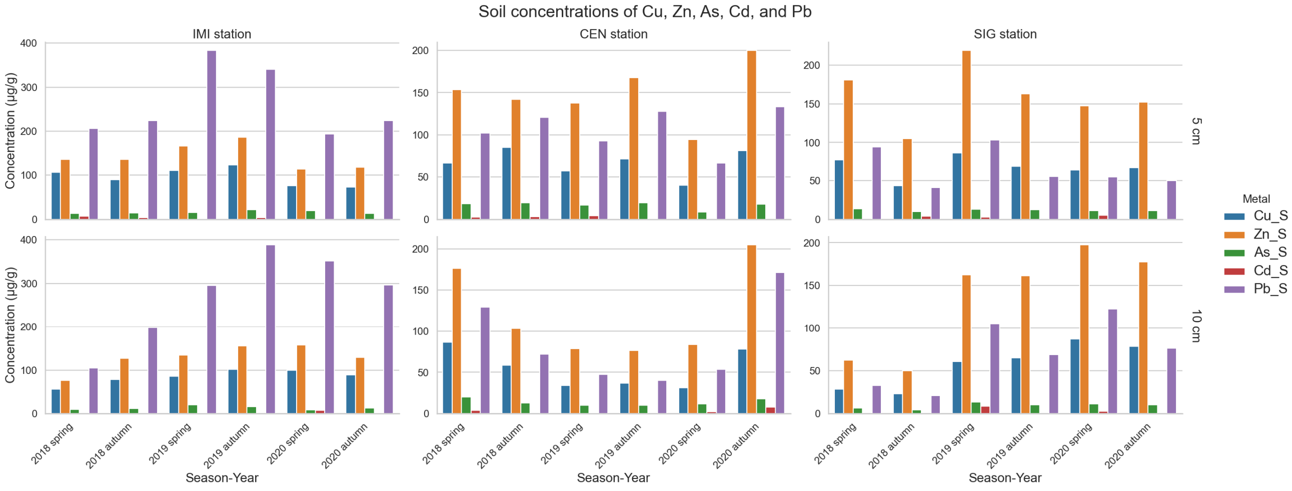

Figure S2. Metals concentration in soil by season, depth and station

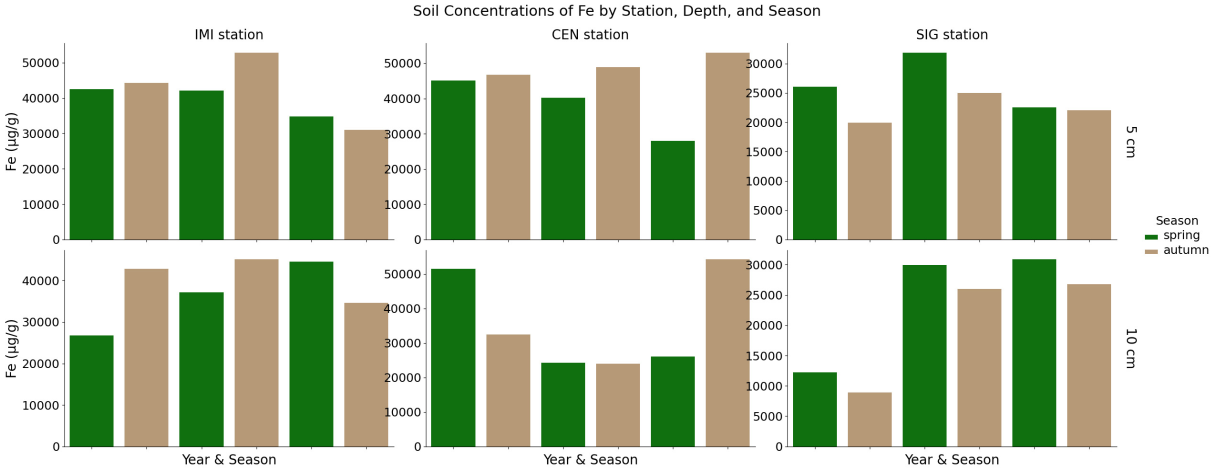

Figure S3. Fe concentration in soil by season, depth and station

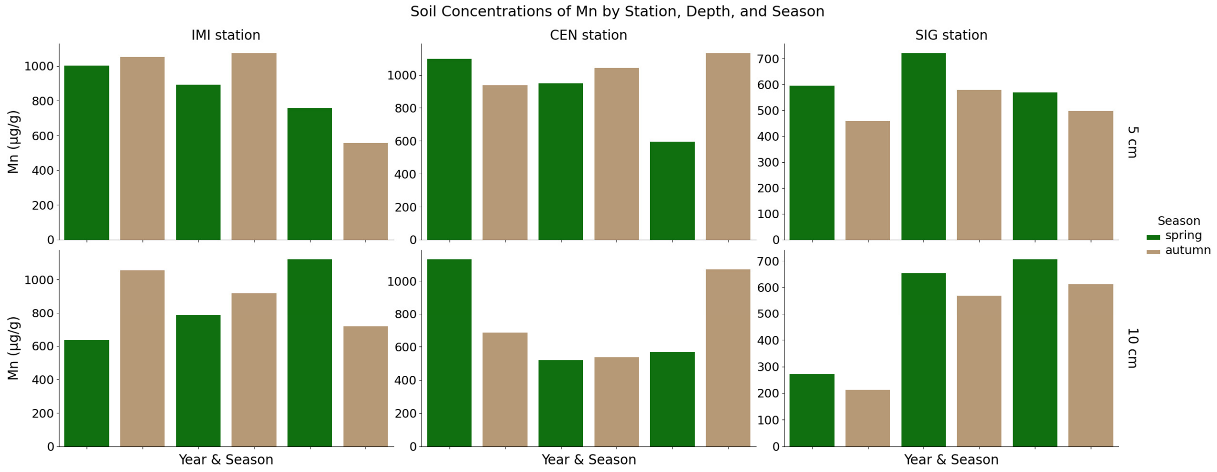

Figure S4. Mn concentration in soil by season, depth and station

Table S1. Correlation analysis for air (PM_{10}) and soil (for different depth) pollutants

pollutant	spearman_r, 5 cm	p_value, 5 cm	spearman_r, 10 cm	p_value, 10 cm
BaA	-0.200	0.704	-0.200	0.704
BaP	-0.486	0.329	-0.314	0.544
BbF	-0.314	0.544	-0.314	0.544
BghiP	-0.600	0.208	-0.314	0.544
BjF	-0.600	0.208	-0.429	0.397
BkF	-0.600	0.208	-0.429	0.397
Flu	-0.714	0.111	-0.657	0.156
Pyr	-0.543	0.266	-0.429	0.397
DahA	-0.714	0.111	-0.314	0.544
IP	-0.600	0.208	-0.429	0.397
Chry	-0.657	0.156	-0.657	0.156
Mn	0.771	0.072	0.943	0.005
Pb	0.943	0.005	0.829	0.042
Zn	-0.257	0.623	-0.257	0.623
Cu	0.714	0.111	0.600	0.208
As	-0.771	0.072	-0.714	0.111
Fe	0.771	0.072	0.886	0.019
Cd	0.883	0.020	0.000	1.000

Table S2. Correlation analysis for air (different time windows) and soil pollutants

pollutant	months	spearman_r, 5 cm	p_value, 5 cm	spearman_r, 10 cm	p_value, 10 cm
As	1	-0.235	0.463	-0.210	0.513
BaA	1	0.063	0.846	0.196	0.542
BaP	1	0.021	0.948	-0.056	0.863
BbF	1	0.056	0.863	-0.021	0.948
BghiP	1	-0.140	0.665	-0.007	0.983
BjF	1	-0.186	0.564	-0.189	0.557
BkF	1	0.028	0.931	-0.077	0.812
Cd	1	0.167	0.667	0.073	0.877
Chry	1	0.035	0.914	0.056	0.863
Cu	1	0.622	0.031	0.308	0.331
DahA	1	-0.175	0.587	-0.371	0.236
Fe	1	-0.028	0.931	0.308	0.331
Flu	1	0.091	0.779	0.399	0.199
IP	1	-0.063	0.846	-0.112	0.729
Mn	1	-0.182	0.572	0.322	0.308
Pb	1	0.825	0.001	0.727	0.007
Pyr	1	0.175	0.587	0.329	0.297
Zn	1	-0.168	0.601	0.217	0.499
As	3	-0.298	0.347	-0.224	0.484
BaA	3	0.538	0.071	0.559	0.059
BaP	3	0.559	0.059	0.734	0.007
BbF	3	0.371	0.236	0.601	0.039
BghiP	3	0.490	0.106	0.503	0.095
BjF	3	0.165	0.609	0.280	0.379
BkF	3	0.357	0.255	0.566	0.055
Cd	3	0.159	0.683	0.109	0.816
Chry	3	0.650	0.022	0.706	0.010
Cu	3	0.531	0.075	0.273	0.391
DahA	3	0.147	0.649	0.280	0.379
Fe	3	-0.371	0.236	0.189	0.557
Flu	3	0.573	0.051	0.629	0.028
IP	3	0.503	0.095	0.706	0.010
Mn	3	-0.392	0.208	0.217	0.499
Pb	3	0.713	0.009	0.573	0.051
Pyr	3	0.490	0.106	0.566	0.055
Zn	3	-0.413	0.182	0.070	0.829
As	6	-0.336	0.285	-0.217	0.499
BaA	6	0.175	0.587	0.427	0.167
BaP	6	0.434	0.159	0.671	0.017
BbF	6	0.357	0.255	0.699	0.011

BghiP	6	0.385	0.217	0.601	0.039
BjF	6	0.312	0.324	0.510	0.090
BkF	6	0.392	0.208	0.615	0.033
Cd	6	-0.084	0.831	0.182	0.696
Chry	6	0.392	0.208	0.559	0.059
Cu	6	0.455	0.138	0.273	0.391
DahA	6	0.273	0.391	0.650	0.022
Fe	6	-0.455	0.138	0.168	0.602
Flu	6	0.524	0.080	0.720	0.008
IP	6	0.308	0.331	0.538	0.071
Mn	6	-0.392	0.208	0.259	0.417
Pb	6	0.713	0.009	0.615	0.033
Pyr	6	0.406	0.191	0.601	0.039
Zn	6	-0.371	0.235	0.140	0.665

Table S3. Correlation analysis for air and soil pollutants for different seasons and depths (for time windows of 6 months for air (PM_{10}) pollutant concentrations)

season	pollutant	spearman_r, 5 cm	p_value, 5 cm	spearman_r, 10 cm	p_value, 10 cm
autumn	As	-0.783	0.013	-0.444	0.232
autumn	BaA	-0.500	0.170	-0.467	0.205
autumn	BaP	-0.550	0.125	-0.433	0.244
autumn	BbF	-0.500	0.170	-0.467	0.205
autumn	BghiP	-0.500	0.170	-0.483	0.187
autumn	BjF	-0.650	0.058	-0.594	0.092
autumn	BkF	-0.636	0.066	-0.533	0.139
autumn	Cd	0.872	0.054	-1.000	
autumn	Chry	-0.500	0.170	-0.467	0.205
autumn	Cu	0.750	0.020	0.427	0.252
autumn	DahA	-0.427	0.252	-0.417	0.265
autumn	Fe	0.517	0.154	0.633	0.067
autumn	Flu	-0.483	0.187	-0.267	0.488
autumn	IP	-0.700	0.036	-0.650	0.058
autumn	Mn	0.517	0.154	0.650	0.058
autumn	Pb	0.900	0.001	0.767	0.016
autumn	Pyr	-0.517	0.154	-0.400	0.286
autumn	Zn	-0.017	0.966	-0.100	0.798
spring	As	-0.368	0.330	-0.450	0.224
spring	BaA	-0.483	0.187	-0.333	0.381
spring	BaP	-0.667	0.050	-0.550	0.125
spring	BbF	-0.650	0.058	-0.483	0.187
spring	BghiP	-0.733	0.025	-0.517	0.154
spring	BjF	-0.700	0.036	-0.550	0.125

spring	BkF	-0.667	0.050	-0.550	0.125
spring	Cd	0.143	0.760	0.595	0.159
spring	Chry	-0.633	0.067	-0.517	0.154
spring	Cu	0.300	0.433	0.083	0.831
spring	DahA	-0.552	0.123	-0.418	0.262
spring	Fe	0.550	0.125	0.333	0.381
spring	Flu	-0.217	0.576	-0.200	0.606
spring	IP	-0.561	0.116	-0.467	0.205
spring	Mn	0.533	0.139	0.250	0.516
spring	Pb	0.650	0.058	0.467	0.205
spring	Pyr	-0.483	0.187	-0.467	0.205
spring	Zn	-0.567	0.112	-0.200	0.606

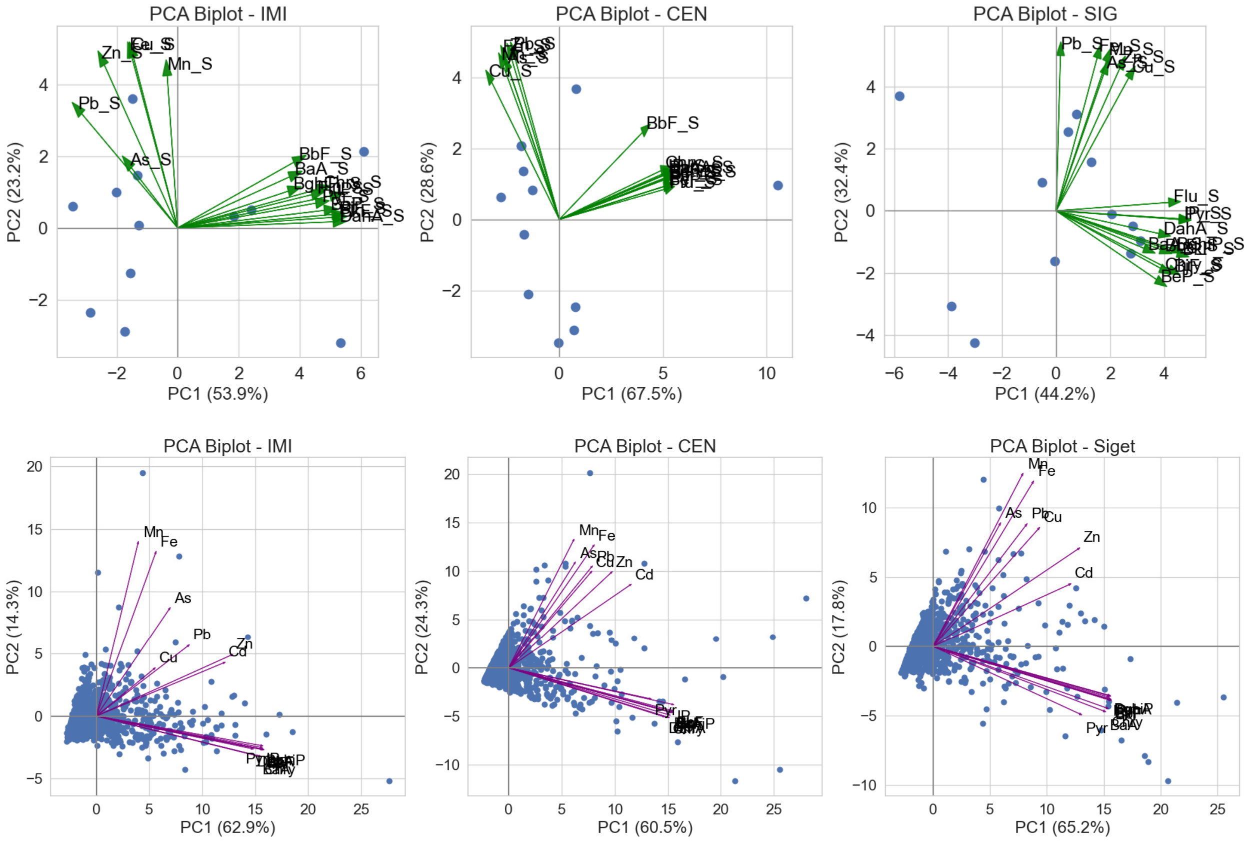

Figure S5. PCA for the soil pollutants (a) and air (PM_{10}) pollutants (b) for different monitoring stations

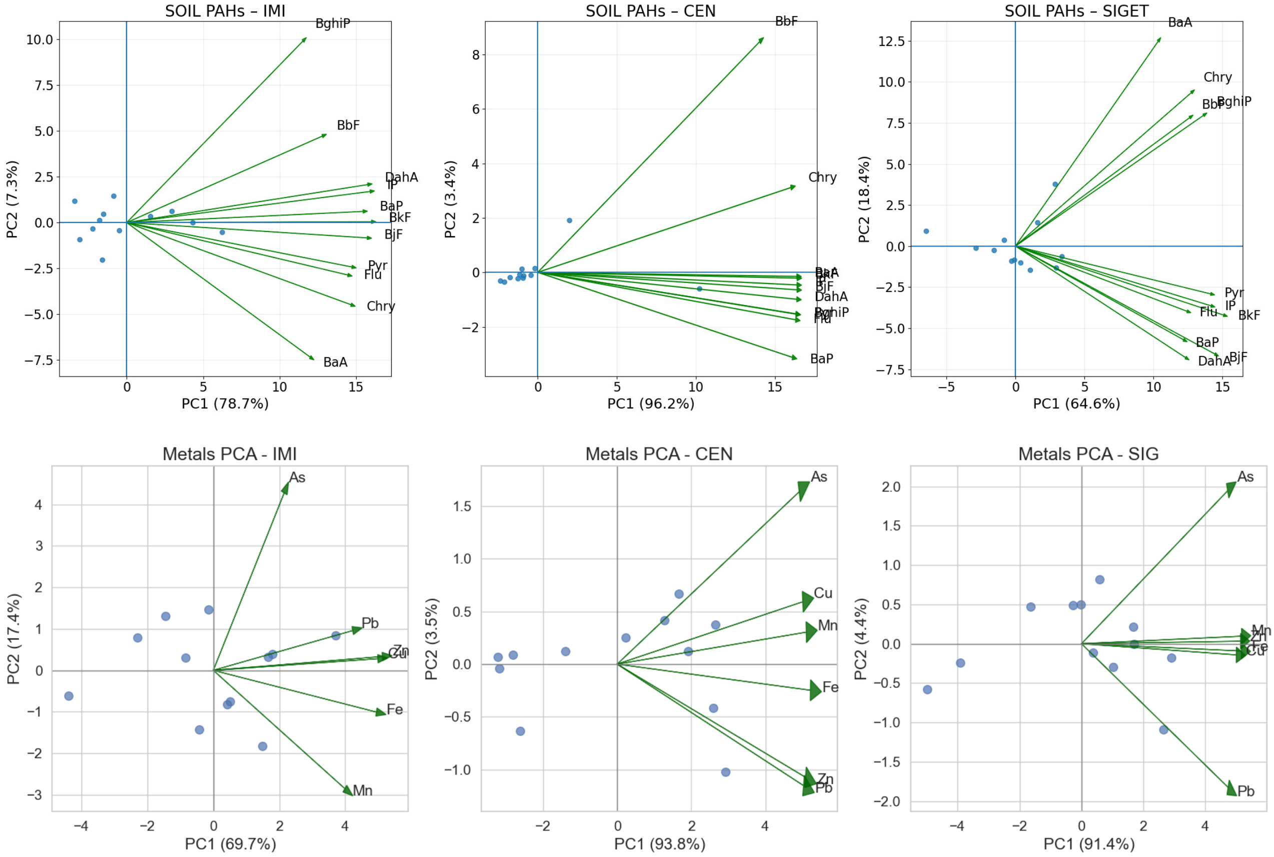

Figure S6. PCA for PAHs and metals in soil samples for different monitoring stations (mean for both depth and periods)

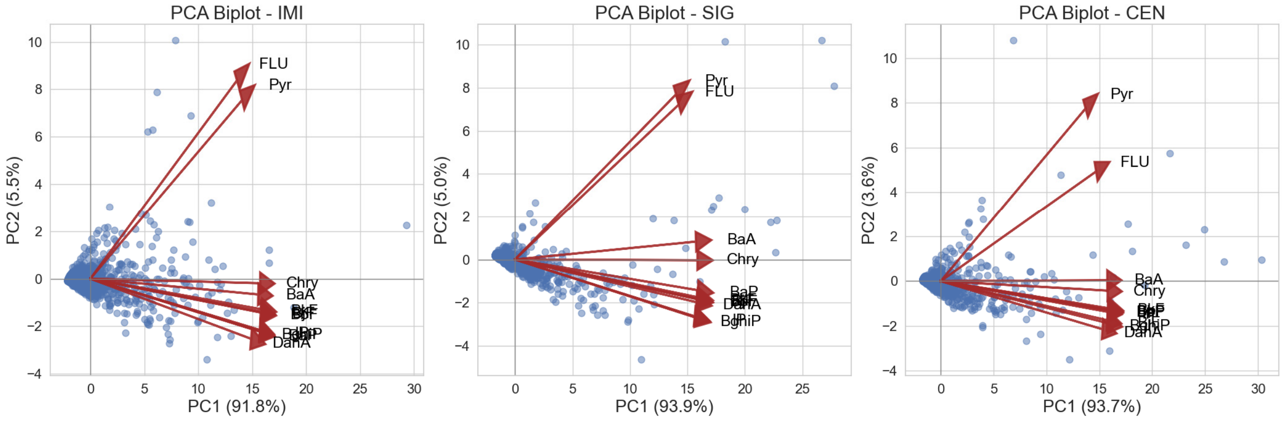

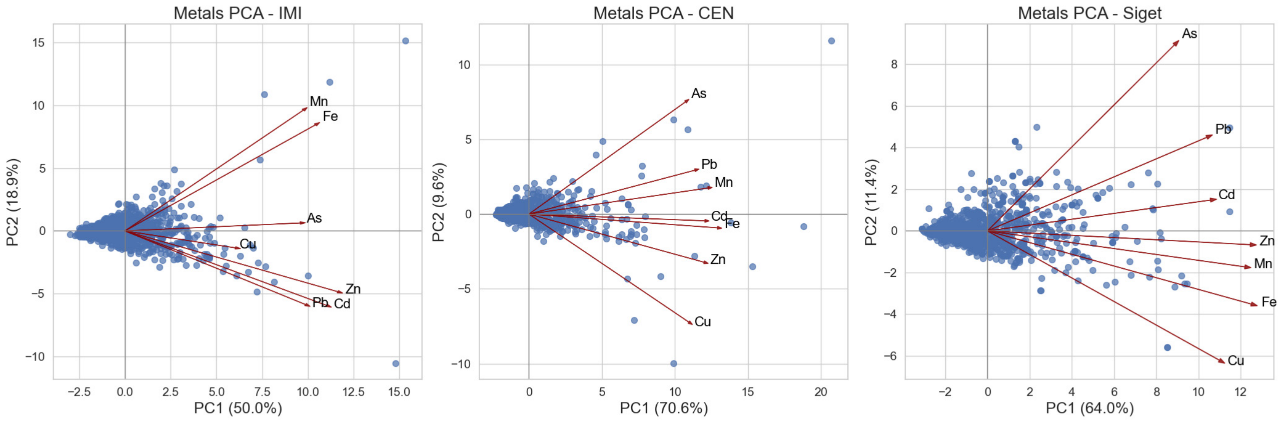

Figure S7. PCA for PAHs and metals air samples for different monitoring stations
